# Critical Shoulder Angle and Its Clinical Correlation in Shoulder Pain

**DOI:** 10.7759/cureus.9810

**Published:** 2020-08-17

**Authors:** Kishore Vellingiri, Prabhu Ethiraj, Arun H Shanthappa

**Affiliations:** 1 Department of Orthopaedics, Sri Devaraj Urs Medical College, Sri Devaraj Urs Academy of Higher Education & Research, Kolar, IND

**Keywords:** critical shoulder angle, glenohumeral osteoarthritis, rotator cuff tears

## Abstract

Introduction

The critical shoulder angle is defined as the angle formed between the plane of the glenoid and the line connecting the most lateral border of the acromion process, as seen on the true anteroposterior radiograph of the shoulder. The purpose of this study was to determine the association between the critical shoulder angle and shoulder pathologies like rotator cuff tears and glenohumeral osteoarthritis. It was also to assess the reproducibility and accuracy of critical shoulder angle values, which were measured with radiographs.

Objective

The aim of the study was to find out the association between the critical shoulder angle and shoulder injuries in a rural population cohort. The secondary aims were to assess clinical and radiological correlations between the critical shoulder angle and the symptomology of shoulder pain.

Materials and methods

Our study analysis was a prospective design conducted at R L Jalappa Hospital & Research Centre, Karnataka, South India. After meeting the inclusion and exclusion criteria, 100 patients were recruited for the study. Forty-five patients had glenohumeral osteoarthritis and 55 patients had a diagnosis of rotator cuff tears. The majority of the patients were male (70%) in both the glenohumeral osteoarthritis and rotator cuff tear groups. The mean critical shoulder angles in the glenohumeral osteoarthritis and rotator cuff tear groups were 30.31 and 33.62, respectively.

Conclusions

Our data aid in demonstrating that glenohumeral osteoarthritis is associated with a significantly narrower critical shoulder angle and wider critical shoulder angles in rotator cuff disease. Further studies, however, should determine whether this association has a cause-and-effect relationship.

## Introduction

The critical shoulder angle is the angle formed between the glenoid fossa plane (the line from the glenoid’s inferior edge to the superior edge of the glenoid) and a line drawn from the inferior edge of the glenoid to the lateral edge of the acromion on a true anteroposterior (Grashey) shoulder radiograph [1]. Wider critical shoulder angle values are usually associated with a full thickness rotator cuff tear whereas small critical shoulder angle values are usually associated with glenohumeral osteoarthritis.

Various biomechanical research shows that shoulder abduction, glenoid compression, and joint shear forces are dependent on the critical shoulder angle, which explains various stress and wear patterns seen in the shoulder joint [2]. Measurement of the critical shoulder angle in proper anteroposterior radiography is needed so as to adjust for the errors that may arise from different positioning of the scapula and acromion.

This present study envisages investigating the incidence of a shoulder injury and the association of the critical shoulder angle in a rural population. We also hope to assess the clinical and radiological correlation of the critical shoulder angle in patients with shoulder pain.

## Materials and methods

This prospective study was conducted by the department of orthopedics at R L Jalappa Hospital & Research Centre, an affiliate of Sri Devaraj Urs Medical College, Tamaka, Kolar, Karnataka, South India, from August 2019 to February 2020 This study was approved by the institutional ethics committee. One hundred patients were included in this study. The inclusion criteria were as follows: age between 40 and 60 years, isolated non-traumatic full-thickness rotator cuff tears, and isolated non-traumatic osteoarthritis. The exclusion criteria included the following: traumatic rotator cuff tears, post-traumatic osteoarthritis, prior surgery, and history of shoulder dislocation or subluxation. Patient demographics, shoulder pain history, and critical shoulder angle assessment using radiological means were recorded. The collected data was encoded and entered into a database. All the quantitative measures were expressed using mean and standard deviation. The significance of the difference in means between the two groups was analyzed using the chi-square test. P-value <0.05 was considered statistically significant.

Measurements

**Figure 1:**
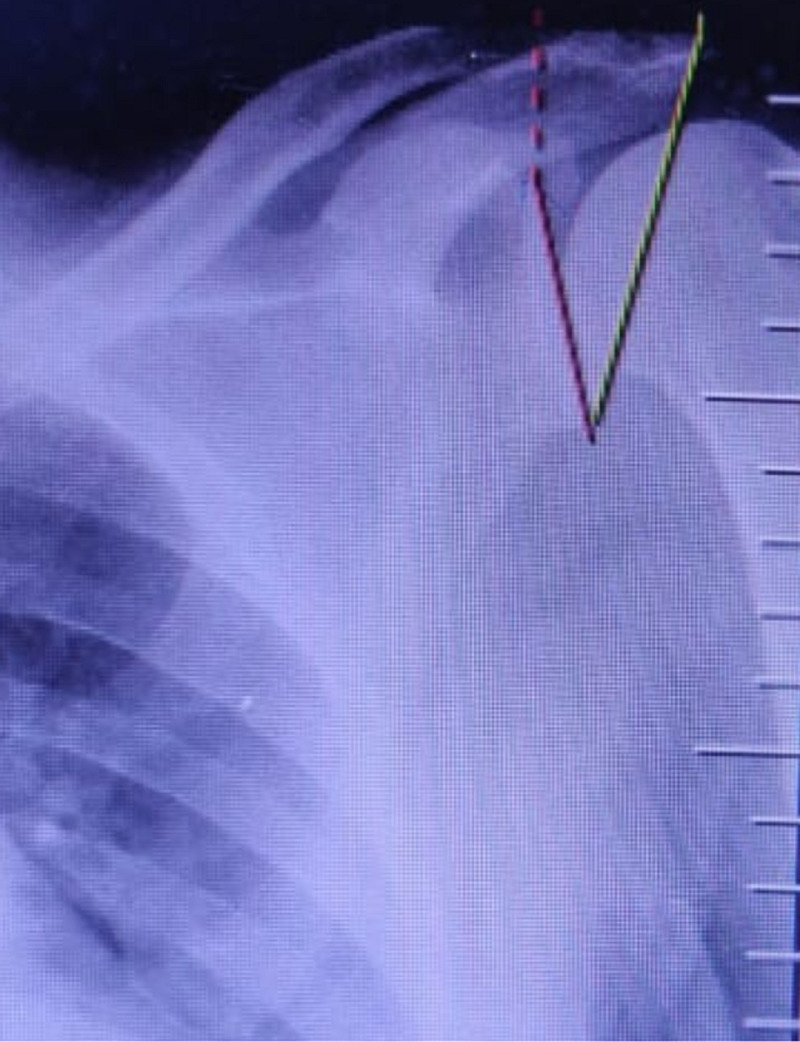
Critical shoulder angle measurement on the AP view AP: anteroposterior

The critical shoulder angle is the angle formed between the glenoid fossa plane (the line from the glenoid’s inferior edge to the superior edge of the glenoid) and a line drawn from the inferior edge of the glenoid to the lateral edge of the acromion on a true anteroposterior (Grashey) shoulder radiograph (Figure 1). An increased critical shoulder angle (35°) is thought to alter deltoid vectors, which results in increased superior shear forces on the rotator cuff muscles [1]. This increased loading of the rotator cuff is a risk factor in developing rotator cuff tears. A reduced critical shoulder angle (30°) is associated with glenohumeral arthritis as the increased compressive forces acting across the glenohumeral joint [1].

## Results

**Figure 2:**
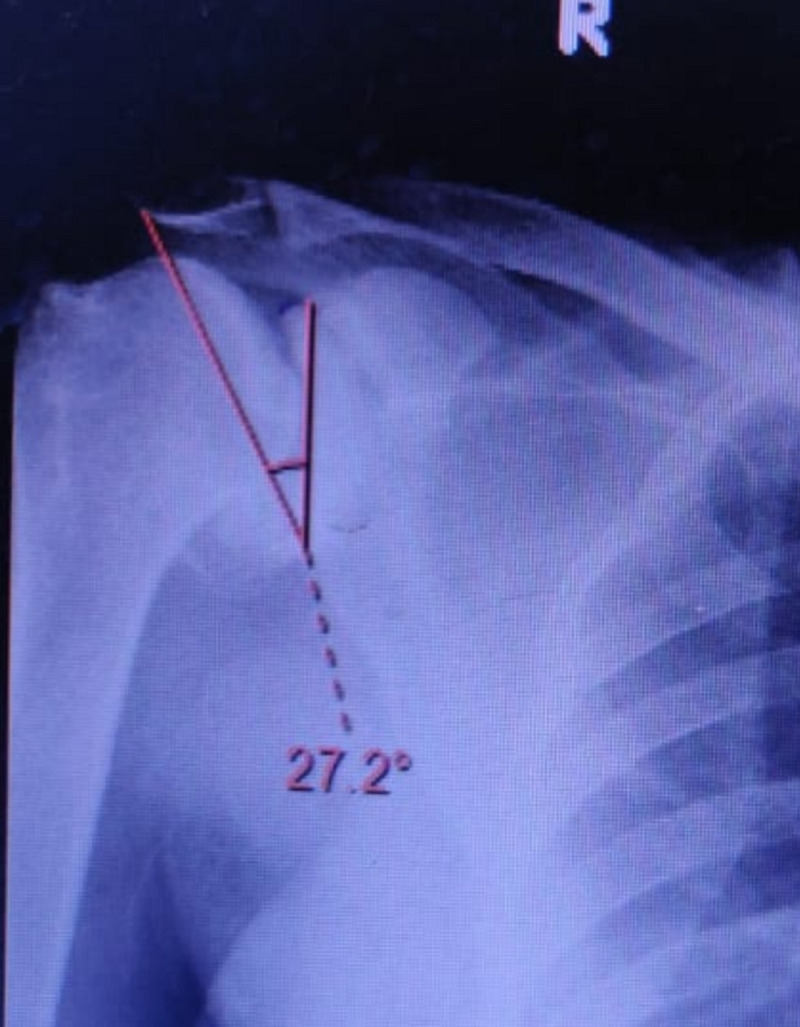
Critical shoulder angle measuring 27.2 degrees in a glenohumeral osteoarthritis patient

**Figure 3:**
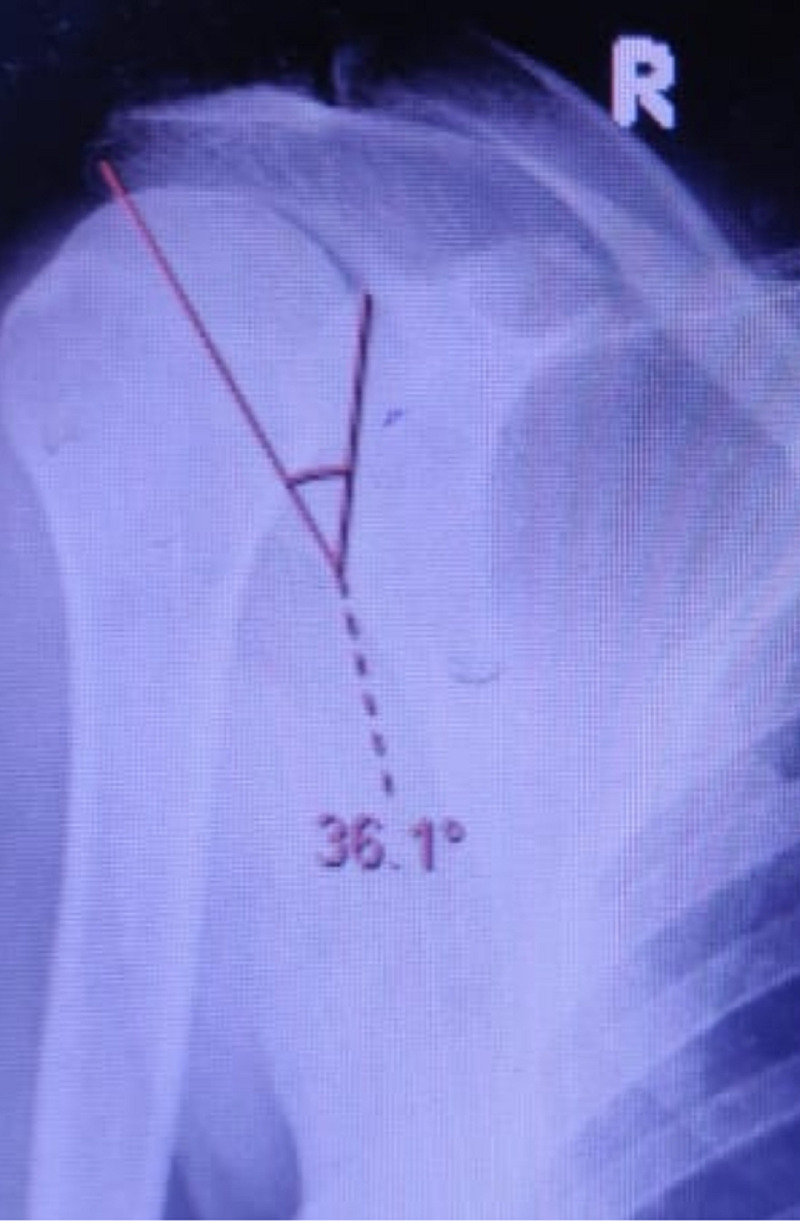
Critical shoulder angle measuring 36.1 degrees in a rotator cuff tear patient

**Figure 4:**
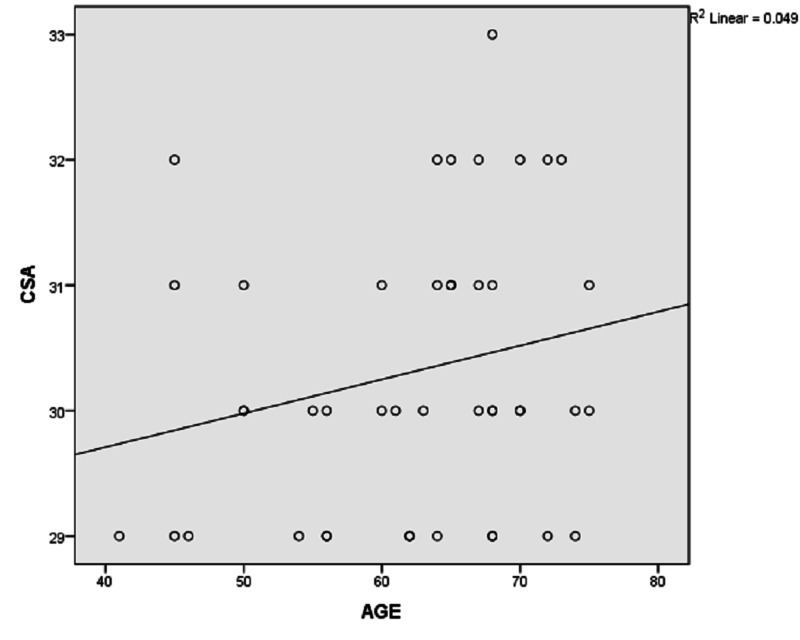
Scatter plot diagram showing a positive correlation between age and critical shoulder angle in the osteoarthritis group CSA: critical shoulder angle

**Figure 5:**
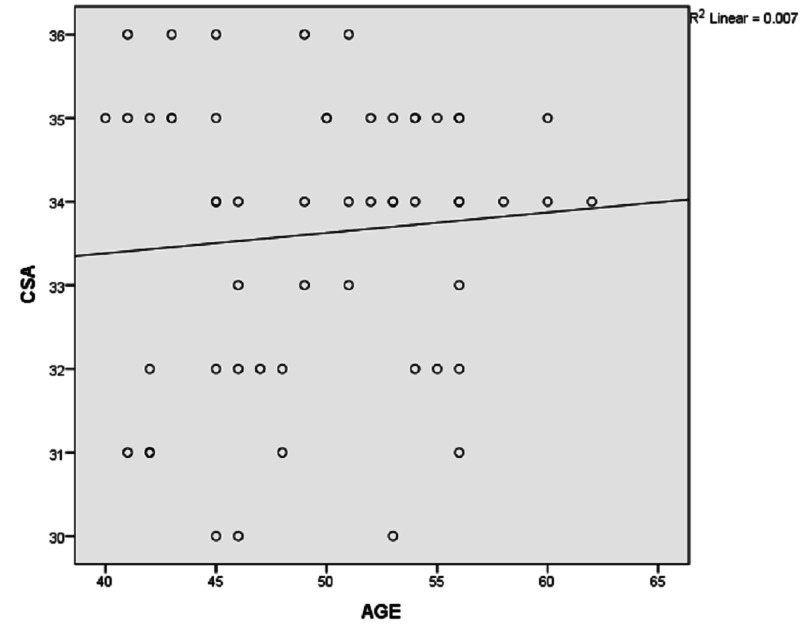
Scatter plot diagram showing a positive correlation between age and critical shoulder angle in the rotator cuff tear group

Our study was done on 100 patients of which 30% were female and the rest of the 70% were males. Thirty-five (35%) were left-sided and sixty-five (65%) were right-sided. The age group distribution was 40-50 years in 34 (34%), 51-60 years in 31 (31%), 61-70 years in 28 (28%), and more than 70 years of age in seven (7%) patients. Most of the patients were male (70%) in both the osteoarthritis and rotator cuff tear groups. Forty-five patients had glenohumeral osteoarthritis and 55 patients were made a diagnosis of rotator cuff tears (Figures 2-3). The mean critical shoulder angles in the osteoarthritis and rotator cuff tears groups were 30.31 and 33.62, respectively. Tables 1-3 show the statistical significance of gender distribution, mean critical shoulder angle, and the correlation between age and the critical shoulder angle, respectively. Figures 4-5 show the scatter plot diagrams.

**Table 1:** Chi-square value and p-value showing that gender distribution is not statistically significant between the osteoarthritis and rotator cuff tear groups OA: osteoarthritis; RCT: rotator cuff tear

		Groups		Chi-square value	P-value
		OA	RCT		
Gender	F	15	15	0.433	0.511
	M	30	40		
Total		45	55		

**Table 2:** Difference in the mean critical shoulder angle between the osteoarthritis and rotator cuff tear groups was found to be statistically significant (p-value <0.01) OA: osteoarthritis; RCT: rotator cuff tear; CSA: critical shoulder angle

	Groups	N	Mean	Std. Deviation	t-value	p-value
CSA	OA	45	30.31	1.125	11.170	<0.01
	RCT	55	33.62	1.705		

**Table 3:** Correlation between age and critical shoulder angle in both the groups Though there is a positive correlation, it is not statistically significant. As age advances, the chance of acquiring the diseases will be high. OA: osteoarthritis; RCT: rotator cuff tear; CSA: critical shoulder angle

Groups	Variables	r-value	p-value
OA	Age & CSA	0.22	0.146
RCT		0.82	0.55

## Discussion

The critical shoulder angle would correlate with the wear of both the rotator cuff tendons or the articular cartilage of the glenohumeral joint. The critical shoulder angle is useful in helping surgeons determine when it is necessary to order a preoperative magnetic resonance imaging study in the evaluation of the rotator cuff. In the original paper, Moor B K et al. suggested that the mean critical shoulder angle was 33° in a healthy shoulder control group, 28° in patients having osteoarthritis, and 38° in the rotator cuff tear group [1]. A larger acromial index was associated with an increased number of tendons torn and anchors used for repair, and it recorded more disability by the Quick Disabilities of the Arm, Shoulder and Hand Outcome Measure and poorer physical health as measured by the Short Form-12 Physical Component Summary score [3]. Critical shoulder measurements from the radiograph demonstrated an excellent interobserver agreement with less variability than critical shoulder angles from magnetic resonance imaging in osteoarthritis patients [4]. Larger critical shoulder angles are associated with increased risk of symptomatic cuff tears, larger cuff tears, and the severity of eccentric osteoarthritis [5]. De Jesus et al. in their study showed that the supplementary scapula position must not be more than 20° of either external or internal rotation [6]. Deviations less than these threshold values were made sure as nonrelevant in previous studies. The advantage of the critical shoulder angle measurement is that it accounts for both glenoid inclination and the lateral extent of the acromion and has been validated in biomechanical models. The lateral acromial roof extension has a higher influence in the pathogenesis of a degenerative rotator cuff tear and concentric osteoarthritis than acromial height or glenoid inclination [7]. The glenoid orientation in the scapulae of shoulders with a full-thickness tear of the rotator cuff was the same as that in shoulders with an intact rotator cuff [8]. The glenoid inclination angle was greater in cadaver shoulders having full-thickness rotator cuff tears (98.6 degrees ) than in shoulders without tears (91.0 degrees ) [9]. Rotator cuff re-tear rates were more in patients with more critical shoulder angles among comparative, non-randomized studies. Based on the previous studies, it is unclear as to whether lateral acromioplasty affects clinical outcomes as a function of a decreased postoperative critical shoulder angle. The structural or mechanical integrity of the lateral deltoid origin was not weakened by arthroscopic lateral acromioplasty and nor did a 5-mm or a 10-mm arthroscopic lateral acromioplasty significantly reduce the deltoid’s failure load [10]. According to Nyffeler et al., the humeral head needed to be in a neutral position or up to a maximum of 20° internal rotation for being included in the definition of a true anteroposterior radiograph [11].

Watson-Jones R [12] and Armstrong JR [13] have implicated the risk factor for developing rotator cuff disease is the acromion’s lateral extension. Nakagawa Y et al. noticed that in primary glenohumeral osteoarthritis, the incidence was around 0.4% in patients with orthopedic complications and 4.6% in patients who suffered from shoulder diseases [14]. The incidence was found to be more among women and older patients above 60 years of age. Minagawa H et al. demonstrated that the prevalence of asymptomatic rotator cuff tear in the general population was 22.1%, of which one-half of all tears were in the 50 years of age, whereas it accounted for two-thirds of those over the age of 60 years [15]. An asymptomatic tear was twice as common as a symptomatic tear. Neer CS in a study identified that total shoulder joint surface replacement had good early outcomes as compared to that of patients treated with hemi-replacement arthroplasty with a Vitallium humeral-head prosthesis in a glenohumeral osteoarthritis condition [16]. Recovery of adequate strength was slow and continued fatigability was noted in the latter part. Hamada K et al. proposed the following causes that lead to cuff-tear arthropathy in massive cuff tear patients: (I) arm elevation in activities of daily living, (II) rupture of the long head of biceps tendon, (III) the abnormal fulcrum of the humeral head against the acromion and the coracoacromial ligament, and (IV) the weakness of external rotation [17]. Moor et al. introduced the critical shoulder angle, which is thought to be predictive of both rotator cuff disease and glenohumeral arthritis [1]. Thus, reviewing the historical acromial indices and measurements is instructive in understanding how the critical shoulder angle is useful as a tool for determining and predicting various abnormalities of the shoulder. Abnormal acromial morphology, especially lateral acromial extension, contributes to rotator cuff disease development by creating altered mechanical vectors that affect both compressive and shear forces. The critical shoulder angle has been linked in developing both rotator cuff tears and osteoarthritis. An increased critical shoulder angle (35°) is thought to result in increased superior shear forces on the rotator cuff muscles due to altered vectors of the deltoid, which may help to predict, and may be a risk factor for developing rotator cuff tears. A decreased critical shoulder angle (30°) is related with glenohumeral arthritis because of an increase in compressive forces across the joint. Published studies have evidence of both supporting and refuting these associations; the conflicting findings may be due to the lack of standardized radiographic methods for measuring the critical shoulder angle and/or measurement errors. Critical shoulder angle and age, two assessable variables, adequately help in the prediction of shoulder pathologies in patients with shoulder complaints. Using the prediction model in primary care may be considered, as severe shoulder pathologies might be detected at an earlier time, thus early adequate treatment by a specialist could be applied.

Limitations of the study

The primary limitation of our study was that it covers a small sample size for this prevalence-based study and was done at a single center. There was no control group in the present study. Prospective longitudinal cohort studies involving a standard and reproducible method of critical shoulder angle measurement are needed to evaluate the true relationship between the critical shoulder angle and shoulder disease.

## Conclusions

Our data aid in demonstrating that glenohumeral osteoarthritis is associated with a significantly narrower critical shoulder angle and wider critical shoulder angles in rotator cuff disease. Further studies, however, should determine whether this association has a cause-and-effect relationship. The measurement of the critical shoulder angle on a radiograph being less prone to interobserver variability than on magnetic resonance imaging and the correlation of wider angles with re-tears of the rotator cuff (hence its ability to predict re-tears) make this study significant. It is especially useful in a developing country like India, where patient affordability is an issue and expensive magnetic resonance imaging could be avoided if osteoarthritis/a rotator cuff tear seems more likely based on clinical and radiographic correlation.
